# Peroxisome Proliferator-Activated Receptor α Activation Protects Retinal Ganglion Cells in Ischemia-Reperfusion Retinas

**DOI:** 10.3389/fmed.2021.788663

**Published:** 2021-12-23

**Authors:** Fei Yao, Xuan Zhang, Xueyan Yao, Xiaohua Ren, Xiaobo Xia, Jian Jiang, Lexi Ding

**Affiliations:** ^1^Eye Center of Xiangya Hospital, Central South University, Changsha, China; ^2^Hunan Key Laboratory of Ophthalmology, Changsha, China; ^3^National Clinical Research Center for Geriatric Disorders, Xiangya Hospital, Changsha, China; ^4^Department of Human Resource, Xiangya Hospital, Central South University, Changsha, China

**Keywords:** peroxisome proliferator-activated receptor α, fenofibric acid, ischemia-reperfusion, neuroprotection, retinal ganglion cell, retinal diseases

## Abstract

**Background and Objective:** Retinal ischemia-reperfusion (IR) leads to massive loss of retinal ganglion cells (RGC) and characterizes several blind-causing ophthalmic diseases. However, the mechanism related to retinal IR is controversial, and a drug that could prevent the RGC loss caused by IR is still lacking. This study aimed to investigate the role of endogenous retinal peroxisome proliferator-activated receptor (PPAR)α and the therapeutic effect of its agonist, fenofibric acid (FA), in IR-related retinopathy.

**Materials and Methods:** Fenofibric acid treatment was applied to the Sprague–Dawley rats with IR and retinal cell line 28 cells with oxygen-glucose deprivation (OGD) (an *in vitro* model of IR). Western blotting, real-time PCR, and immunofluorescence were used to examine the expression levels of PPARα, glial fibrillary acidic protein (GFAP), and cyclooxygenase-2 (COX2). Hematoxylin and eosin (HE) staining, propidium iodide (PI) staining, retrograde tracing, and flash visual-evoked potential (FVEP) were applied to assess RGC injury and visual function.

**Results:** Retinal IR down-regulated PPARα expression *in vitro* and *in vivo*. Peroxisome proliferator-activated receptor α activation by FA promoted survival of RGCs, mitigated thinning of the ganglion cell complex, and decreased the latency of positive waves of FVEPs after IR injury. Further, FA treatment enhanced the expression of endogenous PPARα and suppressed the expression of GFAP and COX2 significantly.

**Conclusion:** Peroxisome proliferator-activated receptor α activation by FA is protective against RGC loss in retinal IR condition, which may occur by restoring PPARα expression, inhibiting activation of glial cells, and suppressing retinal inflammation. All these findings indicate the translational potential of FA in treating IR-related retinopathy.

## Introduction

Retinal ganglion cells (RGCs) are the only retinal neurons that directly project their axons to the central nervous system and perform visual function (1). Many retinal diseases such as acute angle-closure glaucoma, retinal vascular occlusions, and anterior ischemic optic neuropathy can directly or indirectly lead to the irreversible death of RGCs and severely threaten eyesight (2–4). The common pathologic feature among these diseases is retinal ischemia/reperfusion (IR). However, the precise pathways and molecular mechanism related to retinal IR are not well-understood and are controversial. A therapeutic drug to prevent RGC death caused by IR is lacking (5). Accordingly, understanding the pathological mechanism of retinal IR and developing effective therapy are imperative.

Peroxisome proliferator-activated receptor (PPAR)α is a ligand-activated transcription factor and member of the nuclear receptor superfamily. It plays an important part in regulation of lipid metabolism and has anti-inflammatory and antioxidant effects under several pathologic conditions (6–9). In the retina, PPARα is expressed in multiple cell types, including the retinal pigment epithelium (RPE), outer nuclear layer (ONL), inner nuclear layer (INL), and ganglion cell layer (GCL) (10), which is essential for lipid metabolism and neuronal survival in the retina (11).

Previous studies have demonstrated that PPARα expression is down-regulated in the retinas of patients suffering from diabetes mellitus (DM), as well as in the retinas of rodents with diabetic retinopathy or oxygen-induced proliferative retinopathy (10, 12). Knockout of PPARα expression aggravates retinal microvascular damage (12), overexpression of PPARα reduces retinal vascular leakage and retinal inflammation caused by diabetes (10), and alleviates retinal neovascularization in diabetic retinopathy (13). Moreover, Fenofibrate (a specific agonist of PPARα) can alleviate retinal damage by reducing apoptosis of capillary pericytes *via* amelioration of retinal inflammation and oxidative stress (14–16). Qiu et al. showed that PPARα activation by fenofibrate displayed therapeutic effects on age-related macular degeneration induced by lasers in rodents (17).

Taken together, these studies suggest that PPARα is a potential therapeutic target for ophthalmic diseases. However, the relationship between PPARα and ophthalmic diseases characterized by retinal IR is unclear. This study investigated whether PPARα is involved in IR-induced retinal injury (by increasing the intraocular pressure to 110 mmHg for 1 h *via* a saline-perfusion system). We also identified whether PPARα activation has protective effects on RGCs in this condition and explored the underlying mechanism of action.

## Materials and Methods

### Ethical Approval of the Study Protocol

Experiments were undertaken in accordance with the Guide for the Care and Use of Laboratory Animals (National Institutes of Health, No. 80-23, Bethesda, MD, USA). The study protocol was approved by the Animal Research Committee of the Xiangya School of Medicine (Changsha City, China).

### Animals

Female Sprague–Dawley rats (200–250 g; 8 weeks; Slaccas, Changsha, China) were housed in an environment with free access to food and water under a 12-h light–dark cycle. In all procedures, rats were anesthetized with a solution of 2% sodium pentobarbital (80 mg/kg, i.p.; Sanshu, Beijing, China) and xylazine (10 mg/kg, i.p.; Huamu, Beijing, China). Oxybuprocaine hydrochloride (Santen Pharmaceuticals, Tokyo, Japan) was used to anesthetize corneas and tropicamide phenylephrine (Santen Pharmaceuticals) was used to dilate pupils.

### Model of Retinal IR

**Figure 1:**
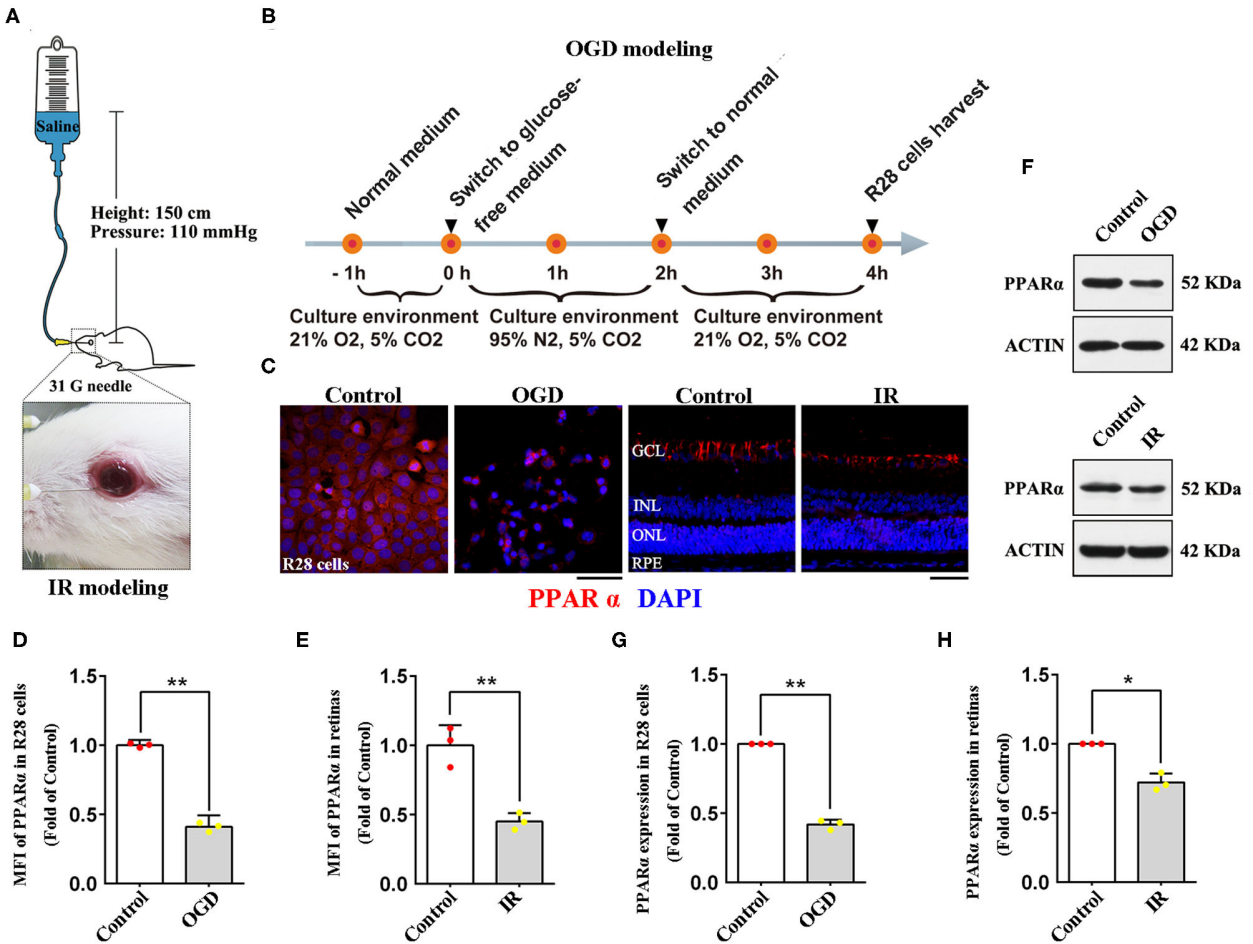
Expression of PPARα in IR-treated retinas (24 h post-modeling) and OGD-treated R28 cells (2 h post-modeling). **(A)** The diagrammatic picture of IR modeling. **(B)** The diagrammatic picture of OGD modeling. **(C)** Representative photomicrographs of immunostained PPARα (red) and nuclei (blue; counterstained by DAPI) in R28 cells and retinas. **(D,E)** Graph showing the mean fluorescence intensity (MFI) of PPARα in R28 cells and retinas. **(F)** The expression of PPARα and actin detected by western blotting in R28 cells and retinas. **(G,H)** Average expression of PPARα semi-quantified by densitometry and normalized to actin expression in R28 cells and retinas. Data are the mean ± SEM; *p < 0.05; **p < 0.01. Scale bar = 50 μm.

Retinal IR injury was induced by increasing the pressure in the anterior chamber *via* the saline-perfusion system described by Tong et al. (18). Briefly, anesthetized animals with anesthetized corneas and dilated pupils were fixed on a heat-preservation countertop, and their underjaws were raised to prevent death from aspiration of saline. Then, 31-G needles were inserted into the anterior chamber and the intraocular pressure was increased gradually to 110 mmHg for 1 h (Figure 1A). Eyes that underwent needle puncture only but not perfusion were regarded as Sham operation (SO) eyes. During and after the procedure, antibiotic eye ointment was used to keep the eyes moist and uninfected. Only rats without saline-leaking or lens injury were included in our study (a total of two rats were excluded because of lens injury and one rat was discarded because of saline-leaking).

### Cell Culture and Oxygen-Glucose Deprivation Model

Retinal cell line 28 (R28) is an adherent retinal precursor cell line derived from neonatal Sprague-Dawley rat retina, which was immortalized by the 12S E1A gene and commonly used to study the function and neuroprotection of RGCs *in vitro* (19). In this study, R28 cells were offered by the Department of Anatomy and Neurobiology (Central South University, Changsha, China). Cells were cultured in low glucose DMEM (11885084; Gibco, Carlsbad, USA) with 10% fetal calf serum (0500; ScienCell, San Diego, USA), 1% L-glutamine (G3126; Sigma, St. Louis, USA), 1% non-essential amino acids (M7145; Sigma, St. Louis, USA), and 1% penicillin-streptomycin (60162ES76; Yeasen, Shanghai, China). To establish the Oxygen-Glucose Deprivation (OGD) model, an *in vitro* model of retinal IR, R28 cells were incubated in a glucose-free DMEM (11966025; Gibco, Carlsbad, USA) with an anoxic environment (95% N_2_, 5% CO_2_) for 2 h, then the cells were cultured in normal medium and reoxygenated (21% O_2_, 5% CO_2_) for 2 h before being examined (Figure 1B).

### PPARα Activation

Fenofibric acid (FA) is a specific agonist of PPARα. *In vivo*, FA (S4527; Selleck Chemicals, Houston, TX, USA) was dissolved in dimethyl sulfoxide (DMSO; Solarbio, Beijing, China) at 10 mg/ml. One hour before IR modeling, animals were injected (i.p.) with FA at 1 ml/kg bodyweight. Treatment was administered once a day (excluding the modeling day) until rats were sacrificed 24 or 72 h after modeling. *In vitro*, 25 μM FA was added to the cell culture medium 1 h before OGD modeling and maintained until the cells were examined.

### Retina Immunofluorescence and Staining

Anesthetized rats were perfused transcardially with pre-cooled phosphate-buffered saline. Intact eyes were enucleated and fixed in FAS eyeball fixative solution (G1109; Servicebio, Beijing, China) for 24 h at room temperature. After fixation, eyeballs were dehydrated in ethanol and embedded in paraffin. Sections (3 μm) were made around the optic nerve for further immunofluorescence staining and hematoxylin and eosin (HE) staining. Dewaxed and antigen-retrieved paraffin retinal slices were applied to immunofluorescence, as described in our previous study (20). Three antibodies (all from Abcam, Cambridge, UK) were used: anti-PPARα (ab215270; 1:100), anti-glial fibrillary acidic protein (GFAP; ab33922; 1:300), and anti-cyclooxygenase 2 (COX2; ab62331; 1:100). Retina HE staining was performed by the Hematoxylin-Eosin/HE Staining Kit (G1120; Servicebio, Wuhan, China) according to the manufacturer's instructions.

### Cell Immunofluorescence and Staining

Retinal cell line 28 cells were fixed in 4% paraformaldehyde for 15 min (G1101; Servicebio, Beijing, China) at room temperature before immunofluorescence staining. The specific procedures and antibodies applied were the same as those used for retinal slices. Besides, propidium iodide (PI; P4170; Sigma, St. Louis, USA) staining and Hoechst staining (A3472; APExBIO, Houston, USA) were applied to detect the survival rate of R28 cells according to the manufacturer's instructions.

### Western Blotting

Freshly isolated retinal tissues were homogenized and lysed in RIPA buffer (P0013; Beyotime, Beijing, China). The protein concentration was quantified by a Bicinchoninic Acid kit (Pierce, Rockford, IL, USA). Western blotting was done as described in our previous study (20) with the following antibodies: anti-PPARα (ab215270; Abcam; 1:1,000), anti-GFAP (ab33922; Abcam; 1:1,000), anti-COX2 (ab62331; Abcam; 1:1,000), and anti-β-actin (GB12001; Servicebio; 1:3,000). The expression levels of PPARα, GFAP and COX2 were quantified by Image-Pro Plus 6.0 (Media Cybernetics, Rockville, MD, USA) and normalized by the densitometry of β-actin.

### Real-Time PCR

Total RNA was isolated from fresh retinal tissues with RNAiso buffer (GB3013; Servicebio, Wuhan, China). Peroxisome proliferator-activated receptor α mRNA was reverse transcribed to cDNA with the Servicebio®RT First Strand cDNA Synthesis Kit (G3330; Servicebio), according to the manufacturer's instructions. Peroxisome proliferator-activated receptor α mRNA expression was quantified using 2 × SYBR Green qPCR Master Mix (G3322; Servicebio). Peroxisome proliferator-activated receptor α mRNA levels were normalized to GAPDH mRNA levels. The following specific primers were synthesized by Servicebio: rat PPARα primers, forward 5′-CATCGAGTGTCGAATATGTGG-3′ and reverse 5′-GCAGTACTGGCATTTGTTCC-3′; rat GAPDH primers, forward 5′-GGAAGCTTGTCATCAATGGAAATC-3′ and reverse 5′-TGATGACCCTTTTGGCTCCC-3′.

### Retrograde Tracing of RGCs

One week before IR modeling, 4% fluorogold (FG; Fluorochrome, Denver, CO, USA) solution was injected into the bilateral superior colliculi of rats (6 mm posterior to the bregma, 1.8 mm lateral to the cranial midline, and 4 mm deep to the cranial surface) to retrograde label RGCs, as described in our previous study (21).

### Counting FG-Labeled RGCs

Flattened retinas were examined by a fluorescence microscope (DM5000 B; Leica, Wetzlar, Germany). Twelve images per retina were taken at 0.85, 2.26, and 3.68 mm (approximately 1/6, 1/2, and 5/6 retinal radius) from the optic disk in superonasal, inferonasal, superotemporal, and inferotemporal quadrants. A double-blind method and Image-Pro Plus 6.0 were used to count the labeled RGCs in each photomicrograph.

### Thickness of the Ganglion Cell Complex (GCC)

The GCC consists of a retinal nerve-fiber layer, GCL and inner plexiform layer, and corresponds to the anatomic distribution of RGCs in the retina (22). To better represent the change in GCC thickness, 12 points of GCC thickness per retinal slice stained by HE were measured by Image-Pro Plus 6.0 according to the following parameters: perpendicular to the surface of the RPE layer as well as ±800, ±1,600, ±2,400, ±3,200, ±4,000, and ±4,800 μm away from the center of the optic nerve (**Figure 3F**).

### Flash Visual-Evoked Potentials

Flash Visual-Evoked Potentials (FVEPs) were obtained using a multifocal electroretinography recorder (GT-2008V-VI; Gotec, Chongqing, China) for functional evaluation of retinas 24 or 72 h after modeling. The stimuli intensity was set to 10.0 cd•s/m^2^, the flash frequency was 1 Hz, and the number of flashes was 64. After light adaptation for 15 min, anesthetized animals were fixed on a special holder with one silver-plate electrode inserted under the skin of the occipital bone (anode), anterior bregma (cathode), and ear (ground electrode), respectively. Then, the FVEP of right and left eyes was recorded in order by a Ganzfeld electrodiagnostic system (Gotec, Chongqing, China). The latency of the first positive wave (P1) and second positive wave (P2) of FVEP was analyzed.

### Statistical Analysis

SPSS 22.0 (IBM, Armonk, NY, USA) was utilized for statistical analyses. Data are the mean ± SEM. The Student's *t*-test and one-way analysis of variance followed by Tukey's *post-hoc* test were used for comparisons between two groups and more than two groups. Statistical significance was set at *p* < 0.05.

## Results

### Retinal IR Down-Regulated PPARα Expression *in vitro* and *in vivo*

To verify whether PPARα is involved in the pathological process of retinal IR, we delineated PPARα expression in R28 cells and retinas after OGD/IR modeling by immunofluorescence analyses and western blotting. Oxygen-glucose deprivation-treated R28 cells showed significant cytoplasmic declination of PPARα expression compared with that in the control group (Figures 1C,D,F,G). In retinas, immunostained PPARα was detected in the GCL, INL, ONL, and the RPE, but most PPARα was expressed in the GCL (Figure 1C). Twenty-four hours after IR modeling, retinal immunolabeling level of PPARα was down-regulated, and this change was manifested mainly in the GCL (Figures 1C,E,F,H). Taken together, these data suggested that retinal IR procedure induced down-regulation of PPARα expression both *in vitro* and *in vivo*; PPARα was likely to participate in the pathological mechanism of retinal IR.

### Protective Effect of PPARα Activation on RGC Survival

**Figure 2:**
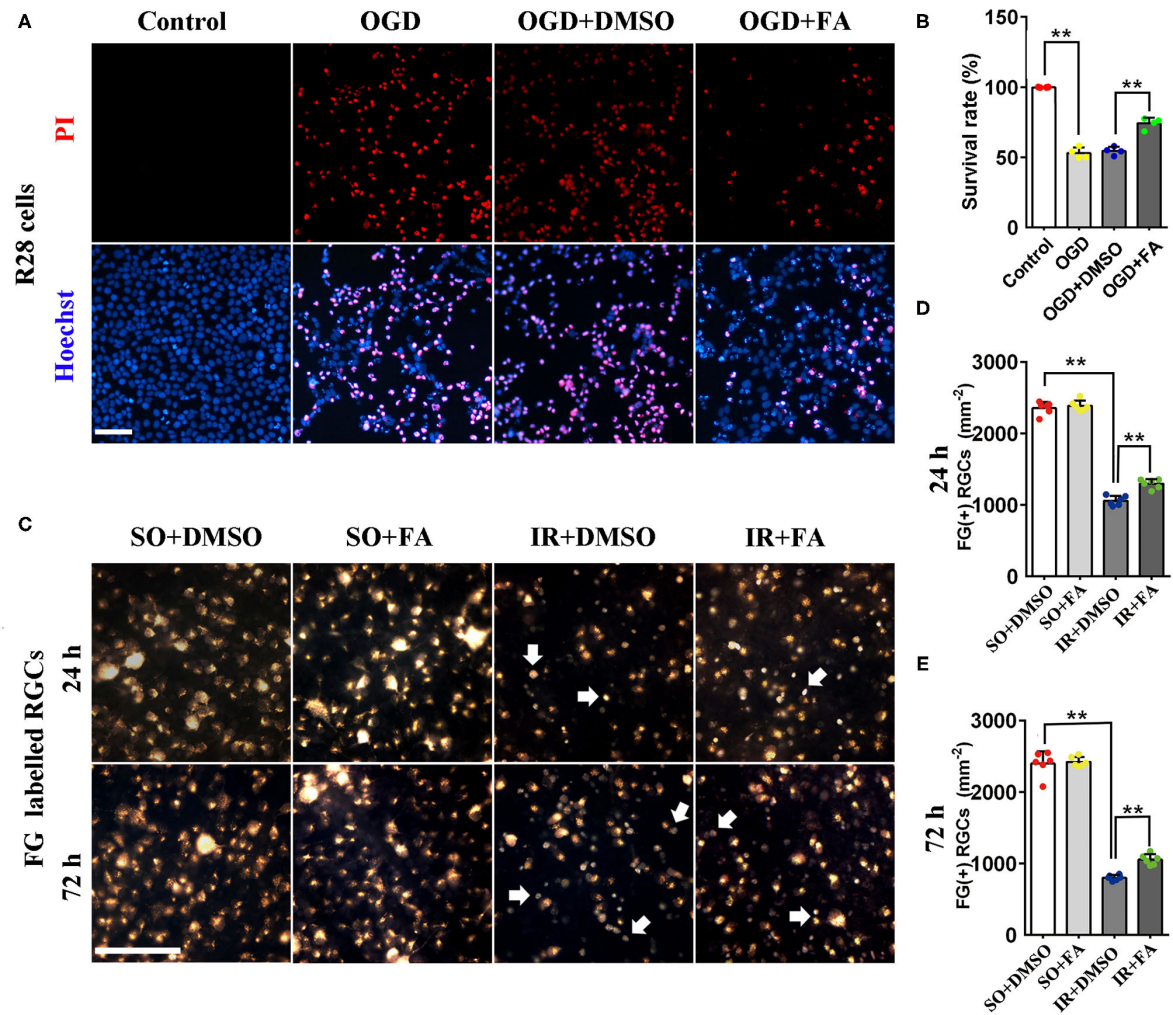
Protective effect of PPARα activation by FA on RGC survival *in vitro* and *in vivo*. **(A)** Representative photomicrographs of R28 cells stained by propidium iodide (PI, dead cells) and Hoechst (total cells). **(B)** The survival rate of R28 cells. **(C)** RGCs labeled by fluorogold (FG) taken at 1/2 retinal radius distances from the optic disk (white arrows, dead RGCs). **(D,E)** The average number of FG-labeled RGCs at 24 h/72 h post-IR modeling. Data are the mean ± SEM; **p < 0.01. Scale bar = 50 μm.

To clarify the role of PPARα in retinal IR, we used FA to activate PPARα and evaluated the effect of PPARα activation on R28 cells and RGC survival after OGD/IR modeling. As shown in Figures 2A,B, OGD modeling led to nearly half of R28 cells death 2 h after modeling, and FA treatment ameliorated OGD-induced cell death significantly (*p* = 0.0001, *n* = 4 per group). *In vivo*, surviving RGCs labeled by FG had a granular appearance with clear borders and golden color, whereas dead RGCs had a smaller volume and lighter color (white arrows, Figure 2C). Photomicrographs identified that dead RGCs appeared only in IR groups. The number of surviving RGCs was decreased markedly in IR rats in comparison with SO rats 24 and 72 h after modeling (*p* = 0.0001 for both, *n* = 6 per group). Moreover, FA treatment was able to increase the number of surviving RGCs (*p* = 0.0001 at 24 h and *p* = 0.002 at 72 h, *n* = 6 per group) (Figures 2D,E; Supplementary Table 1). These results suggested that PPARα activation by FA had a protective effect on IR-induced RGC loss.

### PPARα Activation Mitigated Thinning of the GCC

**Figure 3:**
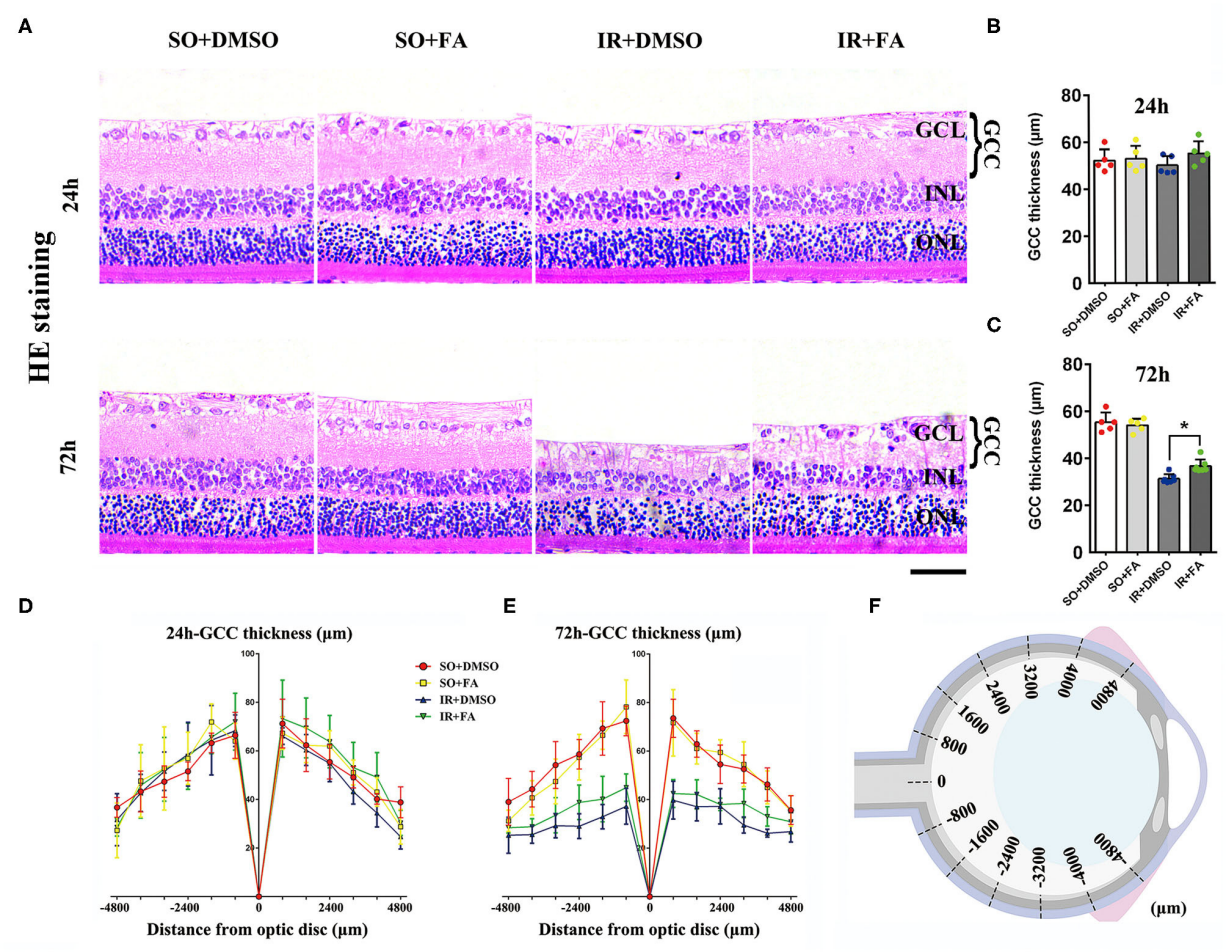
PPARα activation by FA mitigates GCC thinning in IR rats. **(A)** Representative photomicrographs of HE-stained retinal slices taken at 2,400 μm away from the optic nerve at 24 and 72 h post-IR modeling. **(B,C)** Mean thickness of the GCC at 24 h/72 h post-IR modeling. **(D,E)** At 24 and 72 h after modeling, the GCC thickness of rats was measured ± 800, ±1,600, ±2,400, ±3,200, ±4,000, and ±4,800 μm away from the optic nerve. **(F)** The diagrammatic picture of GCC measured in the retina. Data are the mean ± SEM; *p < 0.05. Scale bar = 50 μm.

To further determine the protective effect of PPARα activation on retinal IR, HE staining was used to measure GCC thickness. At 72 h post-modeling, GCC thickness was decreased significantly in IR rats when compared with SO rats (*p* = 0.0001, *n* = 7 in the IR group and *n* = 5 in the SO group). Fenofibric acid treatment efficaciously attenuated GCC thinning (*p* = 0.014, *n* = 7 per group), and all of these changes occurred in the whole retina. However, 24 h after modeling, no obvious difference was found between groups (*n* = 5 per group) (Figure 3; Supplementary Table 1). In summary, FA alleviated the GCC damage induced by IR at 72 h, suggesting that PPARα activation by FA had a protective effect on IR-induced retinal injury.

### Protective Effect of PPARα Activation on Visual Function

**Figure 4:**
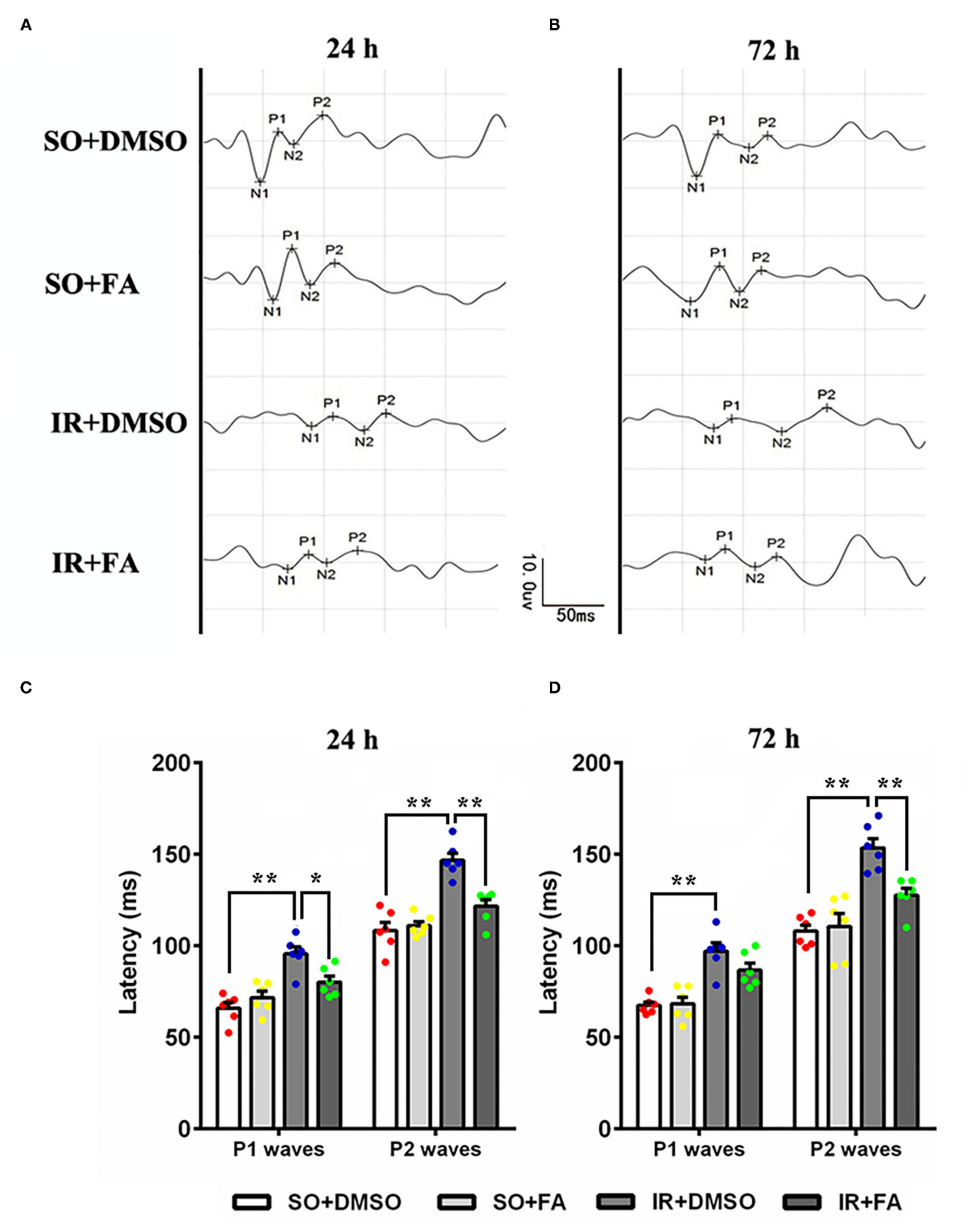
Protective effect of PPARα activation by FA on FVEPs in IR rats. **(A)** Representative images of FVEPs at 24 h post-IR modeling. **(B)** Representative images of FVEPs at 72 h post-IR modeling. **(C)** The latency of the first positive wave (P1 wave) and second positive wave (P2 wave) of FVEPs in rats at 24 h post-IR modeling. **(D)** The latency of the P1 wave and P2 wave of FVEPs in rats at 72 h post-IR modeling. Data are the mean ± SEM; *p < 0.05; **p < 0.01. Scale bar = 10.0 μV and 50 ms.

Moreover, we investigated the protective effect of PPARα activation on visual function. Flash visual-evoked potentials were applied to assess the effect of FA on retinal electrophysiologic activity. The latency of P1 waves and P2 waves was increased by IR at 24 and 72 h after modeling (*p* = 0.0001 for all, *n* = 6 per group), indicating that retinal IR severely affected the conduction function of vision. Furthermore, FA treatment decreased the latency of the P1 wave at 24 h (*p* = 0.021, *n* = 6 per group) and P2 wave at 24 or 72 h after modeling in IR rats (*p* = 0.001 for 24 h and *p* = 0.008 for 72 h, *n* = 6 per group) (Figure 4; Supplementary Table 1), suggesting that PPARα activation by FA ameliorated IR-induced retinal dysfunction.

### FA Increased PPARα Expression *in vitro* and *in vivo*

**Figure 5:**
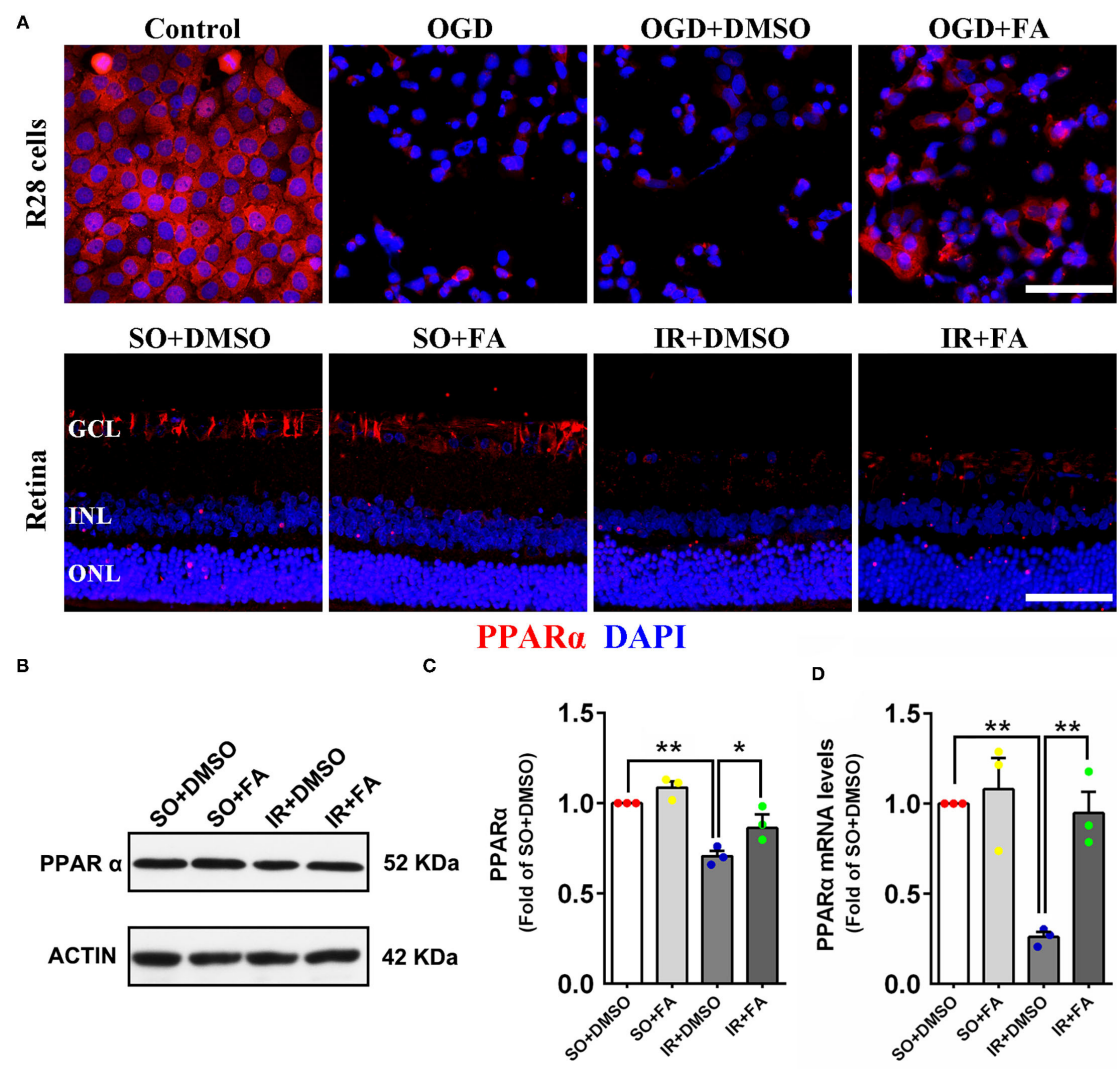
Fenofibric acid increased PPARα expression in OGD-treated R28 cells (2 h post-modeling) and IR-treated retinas (24 h post-modeling). **(A)** Representative photomicrographs of immunostained PPARα (red) and nuclei (blue; counterstained by DAPI) in R28 cells and retinas. **(B)** Retinal expression of PPARα and actin detected by western blotting. **(C)** Average expression of PPARα semi-quantified by densitometry and normalized by actin levels. **(D)** Average expression of PPARα mRNA detected by Real-time PCR and normalized by GAPDH mRNA levels. Data are the mean ± SEM; *p < 0.05; **p < 0.01. Scale bar = 50 μm.

We have demonstrated that retinal IR down-regulated PPARα expression *in vitro* and *in vivo*, and PPARα activation by FA alleviated IR-induced injury and protected visual function. However, the effect of FA on endogenous PPARα expression remains unclear. The immunofluorescence results showed that FA treatment increased PPARα expression in R28 cells and retinas after OGD/IR modeling (Figure 5A; Supplementary Figures 1A,B). Western blotting and Real-time PCR showed that retinal PPARα and mRNA levels were decreased at 24 h post-modeling, while FA treatment effectively reversed these changes (*p* = 0.035 for Western blotting and *p* = 0.0001 for PCR, *n* = 3 per group) (Figures 5B–D). These results suggested that FA as an agonist of PPARα could increase the transcription and translation level of PPARα.

### Activation of PPARα Repressed GFAP and COX2 Expression

**Figure 6:**
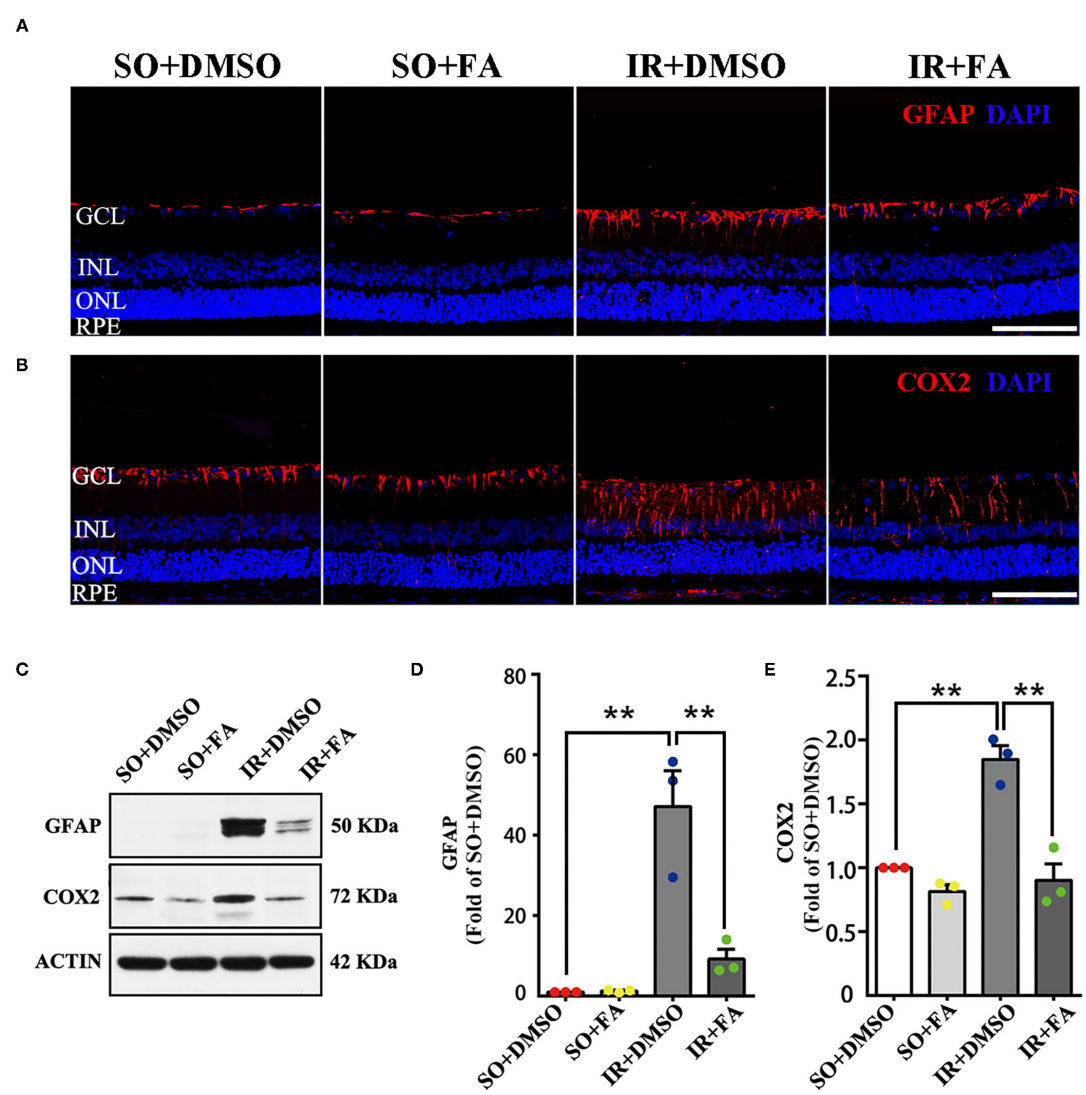
Activation of PPARα by FA repressed GFAP and COX2 expression in the retinas of IR rats. **(A,B)** Representative photomicrographs of immunostained GFAP/COX2 (red) and nuclei (blue; counterstained by DAPI) in retinal sections at 24 h post-modeling. **(C)** Retinal expression of GFAP, COX2, and actin detected by western blotting. **(D,E)** Average expression of GFAP/COX2 semi-quantified by densitometry and normalized by actin levels in retinas. Data are the mean ± SEM; **p < 0.01. Scale bar = 50 μm.

We wished to determine the underlying mechanism of PPARα activation on RGCs in IR rats. Over-activation of glial cells is one of the crucial pathogenic factors after retinal stress injury, up-regulation of GFAP [a marker of glial cells (23)] is regarded as a sensitive non-specific response of glial cells activation (24). In this study, the expression of GFAP was measured in retinas 24 h after IR modeling. Retinal GFAP was expressed mainly in the GCL, and such expression was up-regulated in the GCL of IR rats. However, FA treatment suppressed an up-regulation of GFAP in the GCL (Figure 6A; Supplementary Figure 1C). Consistent with the results of immunofluorescence analyses, western blotting showed that GFAP expression in retinas was also increased after IR modeling (*p* = 0.0001, *n* = 3 per group), FA decreased retinal GFAP level (*p* = 0.0001, *n* = 3 per group) and that there was no difference between rats treated by FA and those treated by DMSO in the SO group (Figures 6C,D).

Cyclooxygenase-2 is an important pro-inflammatory molecule (25). To further elucidate the therapeutic mechanism of FA on IR rats, we measured retinal level of COX2 24 h after IR modeling. Immunostained COX2 was detected in the all layers of the retina. Ischemia-reperfusion modeling increased COX2 level in the GCL and inner plexiform layers significantly. In contrast, FA treatment decreased COX2 level (Figure 6B; Supplementary Figure 1D). Western blotting showed that IR-induced retinopathy up-regulated COX2 level in the retina (*p* = 0.001, *n* = 3 per group), but this level was down-regulated by FA (*p* = 0.0001, *n* = 3 per group. Moreover, there was no distinction between rats treated by FA and those treated by DMSO (Figures 6C,E).

In summary, these results indicated that FA decreased GFAP and COX2 expression in the retina significantly, suggesting that PPARα activation mediated the repression of glial cells activation and suppression of retinal inflammation.

## Discussion

Peroxisome proliferator-activated receptor α is an important lipid-regulating transcription factor. It has been reported that PPARα plays an important part in ophthalmic diseases (14–17). However, the PPARα function in retinal IR is remained unknown. In this study, we first measured PPARα expression in retinal IR condition, and provided evidence that PPARα activation by FA (a PPARα agonist) ameliorated IR-induced RGC injury *in vitro* and *in vivo*. Our results suggested that this beneficial effect was through inhibition of activation of retinal glial cells and suppression of retinal inflammation.

Studies have shown that PPARα is expressed in the retina, kidney, intestine, heart and brain (10, 26–28). Peroxisome proliferator-activated receptor α expression in the retina under chronic ischemic and hypoxic conditions has been studied (10, 12), but PPARα expression under acute ischemia and hypoxia has not. Therefore, we first measured PPARα expression in the retinas of IR rats. Consistent with down-regulation of PPARα expression in chronic ischemic retinopathy [e.g., DM-induced retinopathy (10), oxygen-induced proliferative retinopathy (15)] and in other non-retinal organs injured by IR [e.g., heart (29), kidney (30), and intestine (31)], PPARα expression in the IR retina was decreased 24 h after modeling, indicating that PPARα was involved in the retinal pathological process of IR injury. However, unlike the reduction of PPARα expression in the whole retina caused by DM (10), the decrease in IR-induced PPARα expression was manifested mainly in the GCL. One hypothesis is that IR modeling mainly hindered the blood supply of the inner retina by blocking the central retinal artery and central retinal vein (32). In this case, the GCL was more susceptible to ischemia and hypoxia. This hypothesis could be correct because the thickness of the retinal ONL was not affected by IR (Supplementary Figure 2).

To verify the effect of PPARα in IR-induced retinopathy, we used a specific PPARα agonist, FA, to activate PPARα and ascertain if PPARα plays an important part in this pathologic process. First, we used FG to retrograde-label surviving RGCs in the retina. Retinal ganglion cell loss is a crucial feature of acute angle-closure glaucoma, retinal vascular occlusions, and anterior ischemic optic neuropathy. We found that IR modeling led to a significant loss of RGCs 24 and 72 h after modeling, which is consistent with the pathologic process of the diseases mentioned above. Peroxisome proliferator-activated receptor α activation by FA reduced the RGC loss caused by IR efficaciously, indicating that PPARα had a protective role in this process. Second, we measured retinal GCC thickness [a widely used indicator for detection of RGC loss in clinical diagnoses (33, 34)] after FA treatment to further demonstrate the protective effect of PPARα on RGCs. Our results showed that GCC was thinner than the normal group at 72 h post-modeling, and FA treatment alleviated this structural change in the IR retina. However, a change in GCC thickness 24 h after modeling was not found, indicating that the change in GCC thickness occurred later than RGC death. Third, we detected RGC function. Use of FVEPs is a sensitive method that reflects the function of visual pathways (35). Studies have demonstrated that RGC damage would directly affect the visual conduction function, resulting in extension of the latency of each peak of FVEPs (36, 37). In our study, IR-induced RGC injury also increased the latency of the P1 wave and P2 wave of FVEPs, but FA treatment reduced the prolongation of latency. In summary, PPARα activation by FA alleviated IR-induced damage to the structure and function of RGCs, actions that are consistent with its protective effects in diabetic retinopathy or oxygen-induced proliferative retinopathy (14–17). In addition, our results are also consistent with the studies reported by Bulhak et al. (38) and Ravingerova et al. (39), they both demonstrate that PPARα activation protects myocardium from IR injury. All these results suggest that PPARα activation had a protective role in the retina, and PPARα itself could be a potential target for treatment of IR-induced retinopathy.

Fenofibrate is a synthetic ligand of PPARα and has been used as a hypolipidemic drug for >30 years (40). Fenofibrate is safe and inexpensive, and use of fenofibrate for treating retinal diseases was inspired by two large clinical studies (FIELD and ACCORD) in which researchers reported its robust therapeutic effects on retinopathy in patients with type-2 DM (41, 42). Fenofibric acid is the active metabolite of fenofibrate (43) and retains the function and advantage of fenofibrate while avoiding some off-target effects caused by direct application of fenofibrate [e.g., inhibition of cytochrome-P450 expression or suppression of voltage-dependent K+ channels (44, 45)]. All of these advantages might be conducive to convert FA to a drug for clinical treatment. As for the potential time window for FA treatment of ophthalmic clinical disease (such as acute angle-closure glaucoma, retinal vascular occlusions, and anterior ischemic optic neuropathy), we cannot predict the occurrence of these diseases and administer FA in advance as in this study, so we consider that FA should be administered as soon as these diseases are diagnosed.

We investigated the underlying mechanism of the neuroprotective effect of PPARα activation. Some *in vitro* studies have shown that fenofibrate and FA have a stimulatory effect on elevation of PPARa levels in osteogenic precursor cells (46) and palmitate treated retinal precursor cells (47). Our results demonstrated that FA also increased the expression of PPARα in a retinal IR model, indicating that FA exerts its neuroprotective effect partially relies on up-regulation of PPARα. Glial cells are crucial for maintaining the blood–retinal barrier and RGC survival (24, 48, 49). It has been reported that glial cells may lose their physiologic functions and be activated in disease or injury, and that over-activation of glial cells is related to retinal neurodegeneration (24). Moran et al. demonstrated that PPARα activation can attenuate over-activation of glial cells in an oxygen-induced retinopathy model (15). Hence, we conjectured that PPARα exerted its protective role in an IR model through this mechanism. We found that expression of GFAP (a marker of glial cells) was increased markedly in the GCL after IR modeling, and that FA treatment reduced GFAP expression significantly. Besides, we measured expression of COX2 (an important proinflammatory mediator produced by activated glial cells) because increased COX2 expression can aggravate local inflammation and promote RGC death (50). After IR modeling, COX2 expression increased significantly, and FA treatment attenuated this increase markedly. These data are in accordance with the work of Zhang et al., who also found that activated glial cells could produce COX2 rapidly in injured optic nerves (51). All these results suggested that the therapeutic effect of PPARα activation was due (at least in part) to inhibition of activation of glial cells and reduction of inflammation in the retina. Importantly, the changes in expression of GFAP and COX2 manifested mainly in the inner layer of the retina (especially in the GCL), which provides further evidence that activation of glial cells and inflammation had direct roles in RGC injury.

## Conclusion

In this study, we demonstrated, for the first time, that PPARα is involved in the pathologic process of retinal IR. Peroxisome proliferator-activated receptor α activation by FA ameliorates IR-induced RGC injury and protects visual function, inhibits activation of glial cells and suppresses retinal inflammation. Taken together, these findings suggest that PPARα could be a new target for the treatment of retinal IR-related ophthalmic diseases.

## Data Availability Statement

The original contributions presented in the study are included in the article/Supplementary Materials, further inquiries can be directed to the corresponding author/s.

## Ethics Statement

The animal study was reviewed and approved by Animal Research Committee of the Xiangya School of Medicine.

## Author Contributions

FY wrote the first draft of the paper. XZ, XX, and LD edited the paper. FY, JJ, and LD designed research. FY, XZ, and XY performed research. FY and XR analyzed data. All authors contributed to the article and approved the submitted version.

## Funding

This work was supported by the National Natural Science Foundation of China (81500720 and 82070966 to LD) and the Science and Technology Innovation Program of Hunan Province (2021RC3026 to LD).

## Conflict of Interest

The authors declare that the research was conducted in the absence of any commercial or financial relationships that could be construed as a potential conflict of interest.

## Publisher's Note

All claims expressed in this article are solely those of the authors and do not necessarily represent those of their affiliated organizations, or those of the publisher, the editors and the reviewers. Any product that may be evaluated in this article, or claim that may be made by its manufacturer, is not guaranteed or endorsed by the publisher.
