# Supplementary material for: Peroxisome Proliferator-Activated Receptor α Activation Protects Retinal Ganglion Cells in Ischemia-Reperfusion Retinas
Source: Front Med (Lausanne). 2021 Dec 23;8:788663. doi: 10.3389/fmed.2021.788663 (PMC8732875; doi:10.3389/fmed.2021.788663)
Supplement: Supplementary file 1 [file Data_Sheet_1.docx]

Supplementary Material

# Supplementary Figures and Tables

## Supplementary Figures

##
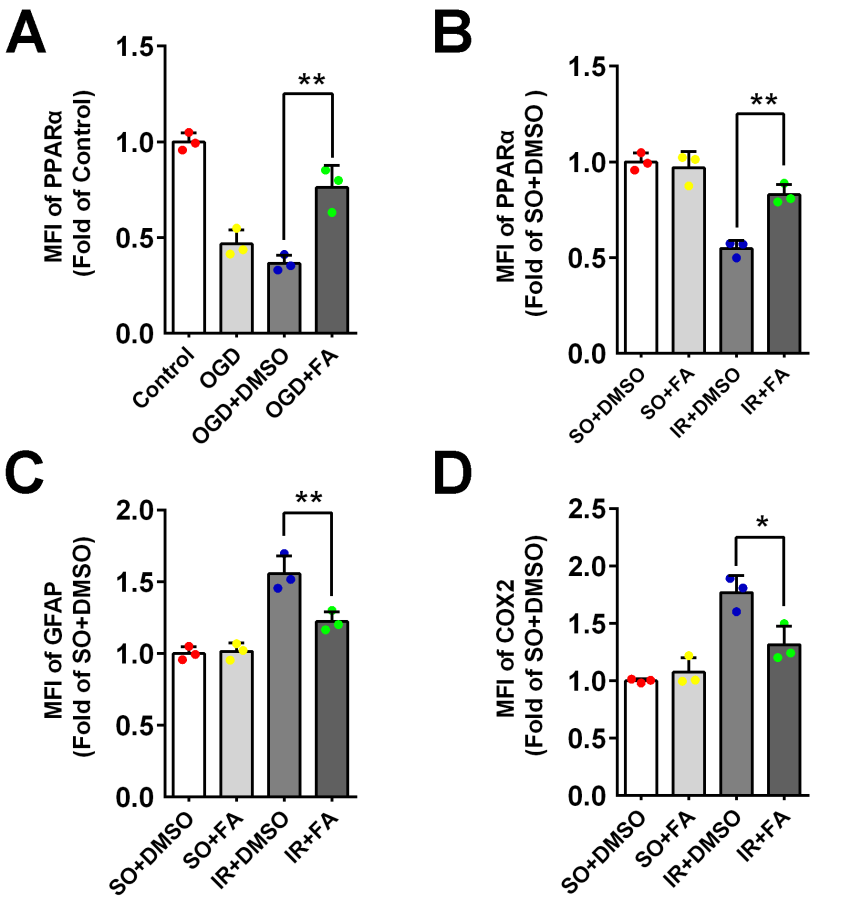


## Supplementary Figure 1. The expression of PPARα, GFAP and COX2 semi-quantified by the mean fluorescence intensity (MFI) in R28 cells and retinas. (A) Graph showing the MFI of PPARα in R28 cells treated by OGD with or without FA treatment. (B-D) Graph showing the MFI of PPARα, GFAP and COX2 in retinas induced by IR with or without FA treatment. COX2, cyclooxygenase 2; DMSO, dimethylsulfoxide; FA, fenofibric acid; GFAP, glial fibrillary acidic protein; IR, ischemia-reperfusion; OGD, oxygen-glucose deprivation; PPARα, peroxisome proliferator-activated receptor α; SO, sham operation. Data are the mean ± SEM; *p < 0.05; **p < 0.01.


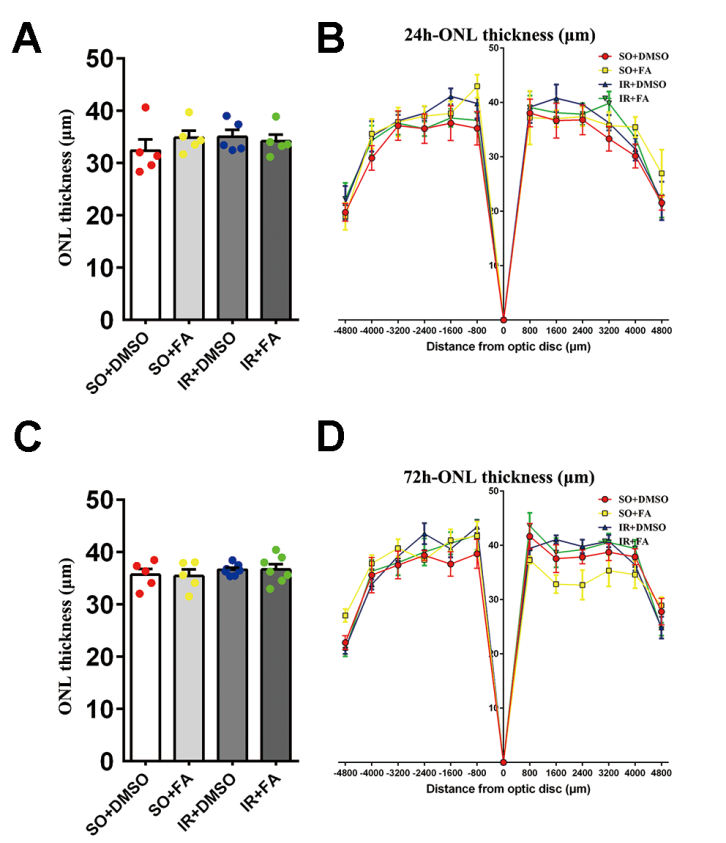


**Supplementary Figure 2.** Outer nuclear layer (ONL) thickness in sham operation (SO) rats and ischemia-reperfusion (IR) rats 24h and 72h after modeling. **(A)** Average thickness of the ONL in rats 24 h after modeling: no significant difference was found between each group (p > 0.05 for all, n = 5 per group). **(B)** At 24 h after modeling, the ONL thickness of rats was measured ± 800, ±1600, ±2400, ±3200, ±4000 and ±4800 μm away from the optic nerve (n = 5 per group). **(C)** Average thickness of the ONL of rats 72 h after modeling: no significant difference was found between each group (p > 0.05 for all, n = 5 in SO groups and n = 7 in IR groups). **(D)** At 72 h after modeling, the ONL thickness of rats was measured ± 800, ±1600, ±2400, ±3200, ±4000 and ±4800 μm away from the optic nerve (n = 5 in SO groups and n=7 in IR groups). DMSO, dimethylsulfoxide; FA, fenofibric acid. Data are the mean ± SEM.

## Supplementary Tables

**Supplementary Table 1**. Changes in retinal parameters after ischemia-reperfusion (IR) modeling and fenofibrate acid (FA) intervention at 24 h and 72 h.

|  | **Parameter**  **(samples per group)** | **SO + DMSO** | **SO + FA** | **IR + DMSO** | **IR + FA** |
| --- | --- | --- | --- | --- | --- |
|  | **GCC thickness (μm)**  n = 5 | 52.2 ± 2.1 | 52.9 ± 2.4 | 50.1 ± 1.7 | 55.1 ± 2.3 |
| **24 h**  **after modeling** | **Surviving RGCs (mm^-2^)**  n = 6 | 2355 ± 35 | 2386 ± 31 | 1058 ± 27^△△^ | 1298 ± 26** |
|  | **P1-wave latency (ms)**  n = 6 | 65.8 ± 3.2 | 71.7 ± 3.3 | 95.6 ± 3.8^△△^ | 80.1 ± 3.2* |
|  | **P2-wave latency (ms)**  n = 6 | 108.3 ± 4.5 | 110.9 ± 2.3 | 146.8 ± 3.8^△△^ | 121.7 ± 3.6** |
|  | **GCC thickness (μm)**  n = 5 or 7 | 55.3 ± 1.9 | 54.1 ± 1.2 | 31.3 ± 0.7^△△^ | 36.7 ± 1.1* |
| **72 h**  **after modeling** | **Surviving RGCs (mm^-2^)**  n = 6 | 2399 ± 70 | 2422 ± 28 | 802 ± 16^△△^ | 1056 ± 31** |
|  | **P1-wave latency (ms)**  n = 6 | 67.3 ± 1.9 | 68.3 ± 3.7 | 97.0 ± 4.6^△△^ | 86.8 ± 3.8 |
|  | **P2-wave latency (ms)**  n = 6 | 108.0 ± 3.3 | 110.7 ± 7.0 | 153.5 ± 5.1^△△^ | 127.7 ± 3.8** |

Data are the mean ± SEM unless stated otherwise.

SO, sham operation; DMSO, dimethyl sulfoxide; GCC, ganglion cell complex; RGC, retinal ganglion cell; P1 wave, first positive wave of flash visual-evoked potentials; P2 wave, second positive wave of flash visual-evoked potentials. ΔΔ p < 0.01 (IR + DMSO group compared with SO + DMSO group); *p < 0.05, **p < 0.01 (IR + FA group compared with IR + DMSO group).
