# Supplementary figures and images for: Peroxisome Proliferator-Activated Receptor α Activation Protects Retinal Ganglion Cells in Ischemia-Reperfusion Retinas
Source: Front Med (Lausanne). 2021 Dec 23;8:788663. doi: 10.3389/fmed.2021.788663 (PMC8732875; doi:10.3389/fmed.2021.788663)

Raw data download links：

https://www.jianguoyun.com/p/DZg_JvoQjpbyCRiCwpsE


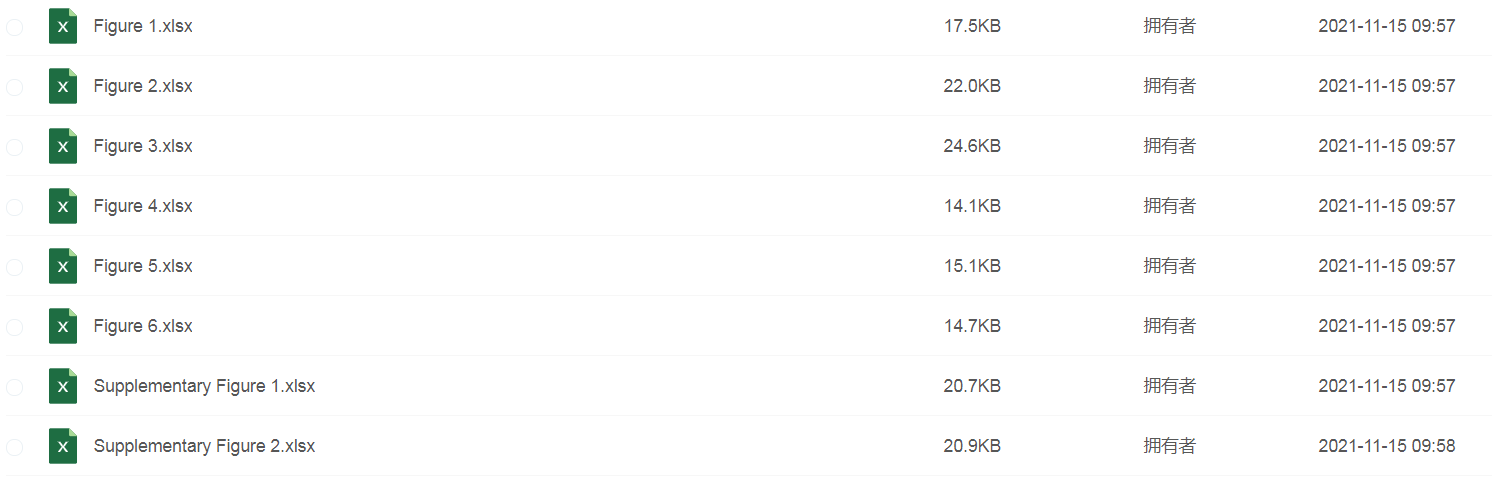


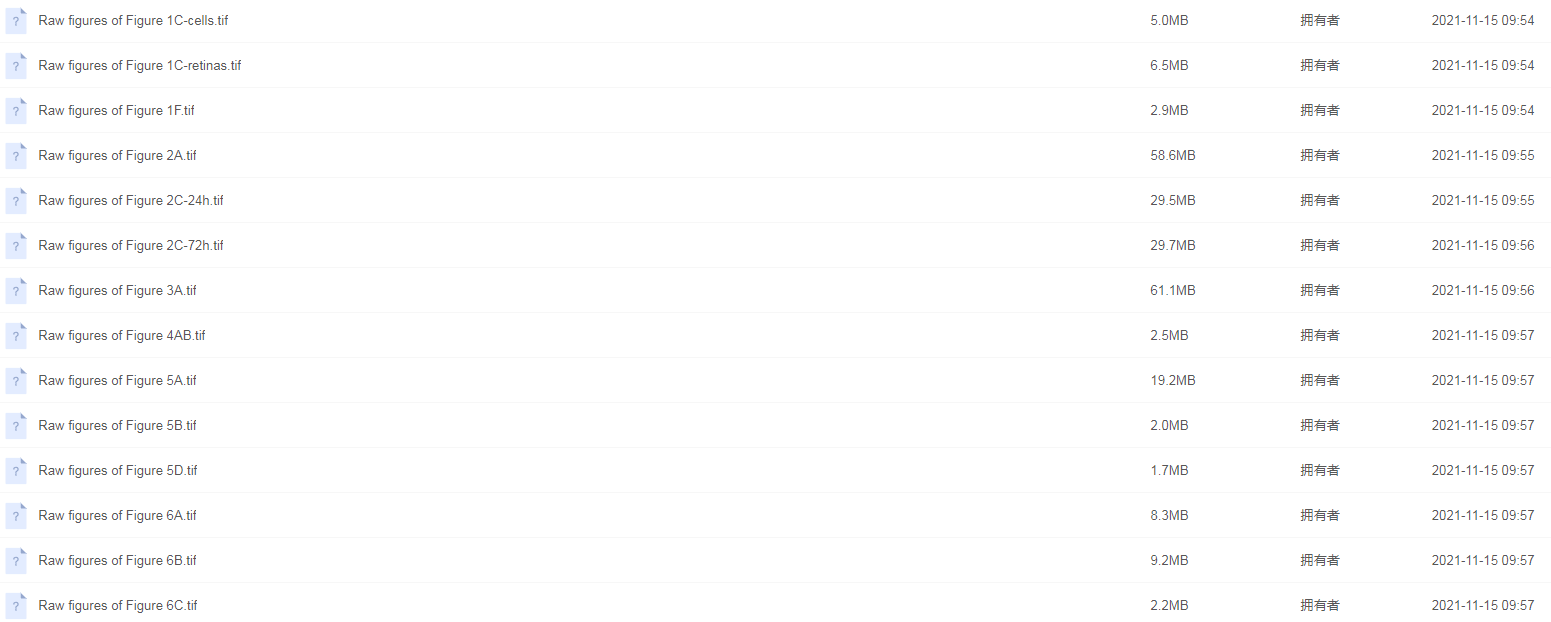

Supplement: Supplementary file 2 [file Data_Sheet_2.DOCX]
